# Spatio-temporal associations of air pollutant concentrations, GP respiratory consultations and respiratory inhaler prescriptions: a 5-year study of primary care in the borough of Lambeth, South London

**DOI:** 10.1186/s12940-021-00730-1

**Published:** 2021-05-07

**Authors:** Mark Ashworth, Antonis Analitis, David Whitney, Evangelia Samoli, Sofia Zafeiratou, Richard Atkinson, Konstantina Dimakopoulou, Sean Beavers, Joel Schwartz, Klea Katsouyanni, on behalf of the STEAM project research group

**Affiliations:** 1 School of Population Health and Environmental Sciences, King’s College London, Guy’s Campus, Addison House, London, SE1 1UL UK; 2 Medical School, National and Kapodistrian University of Athens, Athens, Greece; 3 Population Health Research Institute, St George’s, University of London, Cranmer Terrace, London, SW170RE UK; 4 Environmental Research Group, MRC Centre for Environment and Health, Imperial College, London, UK; 5 Departments of Environmental Health and Epidemiology, Harvard TH Chan School of Public Health, 665 Huntington Avenue, Building 1, Room 1301, Boston, MA 02115 USA

**Keywords:** Air pollutants, Primary care, Respiratory illness, Inhaler prescription, Asthma, COPD

## Abstract

**Background:**

Although the associations of outdoor air pollution exposure with mortality and hospital admissions are well established, few previous studies have reported on primary care clinical and prescribing data. We assessed the associations of short and long-term pollutant exposures with General Practitioner respiratory consultations and inhaler prescriptions.

**Methods:**

Daily primary care data, for 2009–2013, were obtained from Lambeth DataNet (LDN), an anonymised dataset containing coded data from all patients (1.2 million) registered at general practices in Lambeth, an inner-city south London borough. Counts of respiratory consultations and inhaler prescriptions by day and Lower Super Output Area (LSOA) of residence were constructed. We developed models for predicting daily PM_2.5_, PM_10_, NO_2_ and O_3_ per LSOA. We used spatio-temporal mixed effects zero inflated negative binomial models to investigate the simultaneous short- and long-term effects of exposure to pollutants on the number of events.

**Results:**

The mean concentrations of NO_2_, PM_10_, PM_2.5_ and O_3_ over the study period were 50.7, 21.2, 15.6, and 49.9 μg/m^3^ respectively, with all pollutants except NO_2_ having much larger temporal rather than spatial variability. Following short-term exposure increases to PM_10_, NO_2_ and PM_2.5_ the number of consultations and inhaler prescriptions were found to increase, especially for PM_10_ exposure in children which was associated with increases in daily respiratory consultations of 3.4% and inhaler prescriptions of 0.8%, per PM_10_ interquartile range (IQR) increase. Associations further increased after adjustment for weekly average exposures, rising to 6.1 and 1.2%, respectively, for weekly average PM_10_ exposure. In contrast, a short-term increase in O_3_ exposure was associated with decreased number of respiratory consultations. No association was found between long-term exposures to PM_10_, PM_2.5_ and NO_2_ and number of respiratory consultations. Long-term exposure to NO_2_ was associated with an increase (8%) in preventer inhaler prescriptions only.

**Conclusions:**

We found increases in the daily number of GP respiratory consultations and inhaler prescriptions following short-term increases in exposure to NO_2_, PM_10_ and PM_2.5_. These associations are more pronounced in children and persist for at least a week. The association with long term exposure to NO_2_ and preventer inhaler prescriptions indicates likely increased chronic respiratory morbidity.

## Background

The effects of short and long-term exposures to particulate and gaseous air pollution on health have been known for some time [1, 2]. Most studies deal with serious health effects such as increased mortality or hospital admissions associated with both short and long-term exposures [3]. However, a larger proportion of the exposed population are likely to experience less severe effects such as respiratory conditions presenting to primary care.

Few studies have explored adverse health effects of air pollution from a primary care perspective. In a study of particulate pollution (PM_10_) and monthly salbutamol inhaler prescribing in a population of 450,000 patients registered at general practices in north east England, [4], an increase of 10 μg/m^3^ in ambient PM_10_ was found to be associated with a 1% increase in salbutamol prescribing over a 55-month period. Using health insurance data, a study in France covering a population of 260,000 residents of Strasbourg found that 10 μg/m^3^ increases in PM_10_, nitrogen dioxide (NO_2_), and ozone (O_3_) were associated with 7.5, 8.4 and 1% increases in salbutamol sales, respectively, over a 1 year period [5]. In a population of just under one million in Brussels, a study of daily sales of asthma and COPD medication and PM_10_ and NO_2_ residential exposure found no overall association between PM_10_ exposure and medication sales but for NO_2_ exposure, significant associations were found for all age groups except the 85 yr olds; the strongest associations were found in adolescents for whom, a one interquartile range increase in NO_2_ exposure was associated with an 18.7% increase in medication sales, with a 3 week lag, over a 6-year period [6]. None of these studies included clinical data. Based on a study of primary care clinical data over 2 years obtained from the General Practice Research Database between 1992 and 1994, 10th–90th percentile change in PM_10_ particles was associated with a 5.7% increase in upper respiratory tract conditions in adults aged 15–64 years, a 10.2% increase in those aged 65 and over but a non-significant 2.0% increase in those aged 10–14 [7]. Stronger associations were found in the elderly and in the winter months. However, this study did not explore prescribing of inhalers for respiratory conditions.

In order to study associations between air pollution levels and primary care clinical and prescribing data, clinical database linkage is required, linking anonymised clinical and prescribing data with residential air pollution levels. Additionally, appropriate and validated air pollution models are needed to provide estimates of pollution levels at small area level. UK primary care data offers a unique resource to study these associations.

Within the context of the UK MRC funded Project STEAM (Comparative evaluation of Spatio-Temporal Exposure Assessment Methods for estimating the health effects of air pollution), spatiotemporal modelling of several pollutants providing daily data estimating concentrations of pollutants per Lower Super Output Area (LSOA) has been developed [8–10]. We therefore aimed to assess the association between short and long-term pollutant exposures and numbers of primary care respiratory consultations and inhaler prescribing, taking advantage of the U.K. universal registration system in a geographically circumscribed population.

## Methods

### Study area and primary care data

Primary care data were obtained from Lambeth DataNet (LDN), an anonymised dataset containing coded data from all patients registered at general practices in Lambeth, an inner-city south London borough of 26.8 km^2^ [11]. Clinical and prescribing data are available on all current and past registered patients dating back to 2005, a total of 1.2 million patients. Patients are excluded from the dataset if they have an ‘informed dissent’ code in their case-notes, currently accounting for 3.2% of the registered population. Demographic information collected includes data on age, gender and ethnicity. Clinical data includes records of chronic diseases and all medication prescribed. Residential data is also available, down to Lower Super Output Area (LSOA) level, small areas which average 1500 residents in England [12]. At the time of the study, Lambeth consisted of 177 LSOAs.

For the present analysis we used aggregated daily counts, for weekdays only, since primary care data are only available for weekdays, by age group (0–17; 18–64; 65+ years) for the total number of respiratory consultations per LSOA of residence with face to face, telephone, online, administrative consultations and home visits with GP or nurse. The total number of respiratory consultations was stratified into three groups focusing on specific respiratory outcomes for each day over the 5-year period 2009–2013: number of consultations for all asthma episodes, all Chronic Obstructive Pulmonary Disease (COPD) exacerbations and upper respiratory tract infections (URTI) (‘asthma/COPD/URTI consultations’); number of prescriptions for preventer and reliever inhalers (‘inhaler consultations’); total number of respiratory consultations coded for one of the included respiratory conditions &/or issue of a preventer or reliever inhaler (‘all respiratory consultations’). Inhaler prescriptions, both prescribed during a face-to-face consultation or as a repeat prescription were included, since patients often self-manage their respiratory condition through use of the repeat prescription system.

Preventer inhaler prescriptions were defined as those containing long acting beta-agonists (LABA), long acting muscarinic antagonists (LAMA) or inhaled corticosteroids (ICS). Reliever inhaler prescriptions were defined as those containing short acting beta-agonists (SABA) or short acting muscarinic antagonists (SAMA). Read and EMIS medication codes used in the analysis are available on request.

### Air pollution data

Within the STEAM project, we constructed a database of ambient particles with aerodynamic diameter < 10 μm and < 2.5 μm (24-h average PM_10_ and PM_2.5_), Nitrogen Dioxide (NO_2,_ 24-h average) and Ozone (O_3_, 8-h daily maximum) concentrations including all measurements from sites within the M25 orbital motorway during the years 2009–13. Data were obtained from the London Air Quality Network [13], Air Quality England [14] and the Automatic Urban and Rural Network [15]. PM_2.5_ was measured in a smaller number of sites and in order to obtain more data we enhanced the available data based on a generalised additive model (GAM) combination of daily PM2.5 predictions from a regression and a random forest approach based on PM10 and NO_2_ measurements and incorporating seasonality trends, meteorological variables and spatial characteristics. The 10-fold cross-validation adjusted R^2^ of the combined model was 98.9%. The final data included information from 130 sites for NO_2_, 115 for PM_10_, 62 for O_3_ and 104 for PM_2.5_ [16].

Using these data in STEAM, we developed spatio-temporal Land Use Regression (LUR), dispersion models and combinations of these to estimate pollutant concentrations at the postcode centroid level. All postcode centroids within an LSOA were then averaged to produce estimates of LSOA level concentrations. Additionally, for PM_2.5_, we incorporated satellite measurements and applied three machine learning algorithms that were combined in a GAM to produce spatio-temporal concentrations within 1 km × 1 km grids. These models are described in more detail elsewhere [8–10]. Based on simulation studies [8, 9], a GAM model combining predicted pollutant concentrations from the developed spatio-temporal LUR and dispersion models was found to produce the smallest bias on the effect estimates. Daily time series predictions for NO_2,_ O_3_, PM_2.5_ and PM_10_ at LSOA level for Lambeth from this minimum-bias model were used here for the present analysis of the health data.

### Statistical analysis

We used, alternatively, the following outcome variables based on daily counts per LSOA over the study period 2009–13 stratified by age group (0–17; 18–64; 65+ years): (1) ‘asthma/COPD/URTI consultations’, (2) ‘inhaler prescriptions’, (3) ‘all respiratory consultations’.

We investigated the simultaneous short- and long-term effects of exposure to NO_2,_ O_3_, PM_2.5_ and PM_10_ on the outcomes [17, 18]. To assess long-term exposure associations with the outcome, we used the mean pollution concentration of each LSOA for the whole study period, 2009–13 (spatial component of the variability), whilst for short-term effects we used the difference between daily concentrations and the long-term mean for each pollutant per LSOA (temporal component of the variability). We applied a mixed effects, zero inflated negative binomial model, including a random intercept per LSOA, using the NBZIMM library in R [19]. As covariates we used: a) covariates with daily variation (short-term): day of week (6 dummy variables), temperature (as a natural spline with 3 degrees of freedom (3df)) time trend, i.e. a variable numbering sequentially all days in the 2009–2013 period, (natural spline, 6df per year), relative humidity (natural spline, 3df) and b) covariates characterizing each LSOA (long-term): proportion of elderly residents (% aged ≥65 years), Index of Multiple Deprivation 2015 [20]. The number of observations was 1304 days per LSOA. We also considered confounding between pollutants and adjusted for pollutants likely to cause confounding effects, for example, for ozone and PM we adjusted for NO_2_ whilst for NO_2_ effect estimates we adjusted for PM_2.5_.

As a sensitivity analysis, we fitted Poisson models to investigate the short-term associations between daily concentrations of pollutants over the entire sample area and the selected clinical and prescribing data in order to compare their performance with our spatio-temporal models.

## Results

Daily consultation and prescribing data for the study area are summarised in Table 1. Over the study period, the mean number of all respiratory consultations per LSOA, per day was 522.23. Stratification of consultations according to diagnostic code shows that many were not assigned an ‘asthma/COPD/URTI consultation’ code but nevertheless resulted in a prescription for a preventer &/or reliever inhaler.

**Table 1:** Daily respiratory consultation and prescribing data for Lambeth (all LSOAs combined) by age group 2009–13 (aggregated time series for total of 1304 days)

	Age 0–17 years Mean (SD)	Age 18–64 years Mean (SD)	Age > 64 years Mean (SD)	All ages Mean (SD)
**Total respiratory consultations**^a^	123.33 (48.79)	290.47 (74.05)	108.43 (28.04)	522.23 (137.58)
**Consultations for asthma, COPD, URTI**	84.09 (40.20)	133.23 (47.14)	34.75 (12.14)	252.07 (90.49)
**Prescriptions for preventer inhaler only**	5.35 (2.91)	33.38 (9.86)	22.28 (7.31)	61.01 (16.19)
**Prescriptions for reliever inhaler only**	26.74 (10.15)	74.43 (20.69)	27.27 (8.55)	128.43 (34.32)
**Prescriptions for both inhaler types**	15.95 (6.34)	71.16 (18.30)	30.45 (9.70)	117.55 (29.90)

^a^Consultations by any primary care healthcare professional including those where only a preventer or reliever inhaler were issued

The level and variability of air pollution and meteorological values for temperature and humidity are summarised in Table 2 displaying daily, ‘temporal’ variability and ‘spatial variability’. In Table 2, for air pollutants, concentrations are shown, not deviations from a long-term mean as used in the models, to illustrate the data in a clearer way. PM_10_, PM_2.5_ and O_3_ have much higher temporal than spatial variability and only NO_2_, which is largely related to traffic sources, has approximately equal temporal and spatial variability. Temporal and spatial correlation coefficients between air pollutants are summarized in Table 3.

**Table 2:** Means, standard deviations (SD) and quartiles of daily pollutant concentrations and meteorological variables for the whole area of Lambeth, 2009–13 (temporal variability) and for daily pollutant concentrations, per LSOA in Lambeth (177 LSOAs), for 2009–13 (spatial variability)

	Temporal variability					Spatial variability				
	Mean (SD)	1st Quartile	2nd Quartile	3rd Quartile	IQR^a^	Mean (SD)	1st Quartile	2nd Quartile	3rd Quartile	IQR^a^
**24 h NO**_**2**_**(μg/m**^**3**^**)**	50.7 (15.4)	39.1	49.7	61.4	22.3	48.4 (16.3)	36.6	45.1	57.6	21.0
**24 h PM**_**10**_**(μg/m**^**3**^**)**	21.2 (8.8)	15.2	18.6	24.3	9.1	20.8 (2.6)	19.2	20.3	21.1	1.9
**24 h PM**_**2.5**_**(μg/m**^**3**^**)**	15.6 (8.2)	10.3	12.8	17.9	7.6	15.4 (1.2)	14.5	15.0	16.0	1.5
**8 h O**_**3**_ **(μg/m**^**3**^**)**	49.9 (18.5)	37.6	50.2	61.9	24.3	50.7 (4.7)	48.8	52.0	54.3	5.5
**24 h temperature (°C)**	11.0 (5.7)	7.0	11.3	15.3						
**24 h relative humidity (%)**	77.2 (10.1)	70.1	77.8	85.1						

^a^*IQR* interquartile range

**Table 3:** Temporal (daily average over all LSOAS) and spatial (overall 2009–13 mean per LSOA) correlation coefficients for the pollutants

	Temporal correlations				Spatial correlations			
	O_3_	NO_2_	PM_10_	PM_2.5_	O_3_	NO_2_	PM_10_	PM_2.5_
**O**_**3**_	1				1			
**NO**_**2**_	−0.60	1			−0.55	1		
**PM**_**10**_	−0.19	0.55	1		−0.83	0.70	1	
**PM**_**2.5**_	−0.29	0.61	0.96	1	−0.80	0.58	0.75	1

Table 4 shows the % change in the number of ‘all respiratory consultations’ and ‘asthma/COPD/URTI consultations’ following short-term (lag 0) and long-term increase in exposure to each pollutant equal to an interquartile range (IQR) increase. Following increases of short-term exposure to PM_10_, NO_2_ and PM_2.5_ the number of all daily respiratory and asthma/COPD/URTI consultations’ increase for all age groups and reach statistical significance with few exceptions; the largest increase in consultations is generally observed for children: 3.04% (95% confidence intervals, 2.41, 3.67) (‘all respiratory consultations’) and 3.38% (95%CIs 2.65, 4.11) (‘asthma/COPD/URTI consultations’) for PM_10_ per IQR; also 2.37% (95%CIs 1.47, 3.28) and 1.37% (95%CIs 0.34, 2.40) respectively per IQR for NO_2_. However, a short-term increase in O_3_ exposure is associated with a significant decrease in the number of consultations. In contrast, there is no association between long-term exposures to PM_10_, PM_2.5_ and NO_2_ and consultation rates in adults; in children increased long-term exposure to NO_2_ and PM_2.5_ is associated with lower consultation rates. Long-term increase in O_3_ exposure is not associated with a change in the number of consultations, with the exception of a 20% increase in the number of all respiratory consultations for the 65+ years age group.

**Table 4:** Change in the number of respiratory consultations (% and 95 Confidence Intervals (CI)) associated with an interquartile increase in short- (daily, ‘lag 0’) and long- (2009–2013) term NO_2_, PM_10_, PM_2.5_ and O_3_ concentrations. Results from mixed effects zero-inflated negative binomial model adjusting for day of the week, temperature, relative humidity, time trend, proportion of elderly residents and index of multiple deprivation

Air pollutant	Outcome variable: change in consultations	All ages	0–17 years	18–64 years	65 + years
**NO**_**2**_	**Short term, % change (95% CI)**				
	Total respiratory consultations	1.16 (0.69, 1.63)	2.37 (1.47, 3.28)	1.16 (0.56, 1.77)	2.16 (1.20, 3.13)
	Consultations for asthma, COPD, URTI	0.92 (0.25, 1.59)	1.37 (0.34, 2.40)	0.52 (−0.34, 1.39)	1.90 (0.44, 3.38)
	**Long term, % change (95% CI)**				
	Total respiratory consultations	2.15 (− 3.76, 8.43)	−7.89 (− 14.62, −0.63)	4.85 (− 1.43, 11.52)	7.11 (− 1.83, 16.88)
	Consultations for asthma, COPD, URTI	0.83 (− 7.57, 9.99)	−8.51 (− 15.96, − 0.40)	5.59 (− 3.34, 15.35)	5.21 (− 9.79, 22.71)
**PM**_**10**_	**Short-term % change (95% CI)**				
	Total respiratory consultations	1.01 (0.68, 1.34)	3.04 (2.41, 3.67)	0.79 (0.37, 1.22)	1.06 (0.40, 1.72)
	Consultations for asthma, COPD, URTI	1.98 (1.50, 2.46)	3.38 (2.65, 4.11)	1.36 (0.75, 1.98)	1.40 (0.39, 2.41)
	**Long-term % change (95% CI)**				
	Total respiratory consultations	0.18 (−2.95, 3.42)	−1.18 (−5.15, 2.95)	0.79 (− 2.48, 4.17)	0.92 (−3.72, 5.79)
	Consultations for asthma, COPD, URTI	0.24 (−4.31, 5.00)	−0.91 (−5.35, 3.73)	1.67 (− 3.02, 6.58)	− 1.26 (−9.07, 7.23)
**PM**_**2.5**_	**Short-term % change (95% CI)**				
	Total respiratory consultations	0.19 (−0.12, 0.51)	1.40 (0.81, 2.00)	0.02 (−0.39, 0.43)	0.35 (−0.28, 0.97)
	Consultations for asthma, COPD, URTI	0.78 (0.33, 1.24)	1.61 (0.92, 2.30)	0.36 (−0.22, 0.95)	0.66 (−0.29, 1.62)
	**Long-term % change (95% CI)**				
	Total respiratory consultations	−2.41 (−7.85, 3.35)	−8.42 (−14.85, −1.49)	1.21 (−4.65, 7.43)	−2.85 (− 10.77, 5.78)
	Consultations for asthma, COPD, URTI	−3.52 (− 11.27, 4.91)	−9.51 (− 16.59, − 1.82)	2.16 (−6.20, 11.25)	− 7.38 (− 20.17, 7.45)
**O**_**3**_	**Short-term % change (95% CI)**				
	Total respiratory consultations	− 2.62 (− 3.26, − 1.98)	−5.01 (−6.18, − 3.83)	−2.69 (− 3.52, − 1.85)	− 3.38 (− 4.65, − 2.10)
	Consultations for asthma, COPD, URTI	−2.87 (− 3.78, − 1.95)	− 4.58 (− 5.93, − 3.20)	−2.27 (− 3.46, − 1.06)	−1.95 (− 3.92, 0.06)
	**Long-term % change (95% CI)**				
	Total respiratory consultations	2.18 (− 2.83, 7.44)	5.12 (−1.43, 12.11)	− 0.09 (− 5.19, 5.28)	3.76 (− 3.63, 11.71)
	Consultations for asthma, COPD, URTI	5.95 (− 16.53,9.58)	5.83 (− 1.52, 13.73)	2.03 (− 5.32, 9.95)	20.21 (5.88, 36.49)

Table 5 shows the % change in the number of inhaler prescriptions overall and for preventive and reliever inhalers separately following short-term or long term exposure to each pollutant. The number of prescriptions for both types of inhalers increase by 2.14% (95%CIs 1.54, 2.75) after short-term increase in NO_2_ exposure and by 0.84% (95%CIs 0.42, 1.26) after short-term increase in PM_10_ exposure. There is no significant association with short-term exposure changes to PM_2.5_ and O_3_. An increase in long-term exposure to NO_2_ is associated with an increase of 8.11% (95%CIs 1.03, 15.69) in preventer inhaler prescriptions only, whilst an increase is also observed for prescriptions of preventer inhalers following long-term increases in exposure to PM_10_ and PM_2.5_ as well, although not reaching the nominal level of statistical significance. A change in long-term exposure to O_3_ is not associated with significant changes in the number of prescriptions.

**Table 5:** Change in the number of inhaler prescriptions (% and 95 Confidence Interval (CI)) associated with an interquartile increase in short- (daily) and long- (2009–2013) term NO_2_, PM_10_, PM_2.5_ and O_3_ concentrations. Results from mixed effects zero-inflated negative binomial model adjusting for day of the week, temperature, relative humidity, time trend, proportion of elderly residents and index of multiple deprivation

	% change (95% CI)							
	NO_**2**_: Short-term	NO_**2**_: Long-term	PM_**10**_: Short-term	PM_**10**_: Long-term	PM_**2.5**_: Short-term	PM_**2.5**_: Long-term	O_**3**_: Short-term	O_**3**_: Long-term
**Preventive inhaler**	2.07 (1.31, 2.85)	8.11 (1.03, 15.69)	0.75 (0.22, 1.29)	2.12 (−1.55, 5.92)	− 0.01 (− 0.51, 0.50)	3.59 (−3.03, 10.67)	− 3.50 (− 4.53, −2.46)	−3.52 (− 8.93, 2.21)
**Reliever inhaler**	2.10 (1.42, 2.78)	1.44 (−4.34, 7.57)	0.77 (0.31,1.24)	− 0.55 (− 3.61, 2.61)	0.01 (− 0.44,0.46)	−3.04 (− 8.36, 2.57)	−3.71 (− 4.61, − 2.80)	1.53 (− 3.37, 6.68)
**Any inhaler**	2.14 (1.54, 2.75)	3.01 (− 2.90, 9.27)	0.84 (0.42, 1.26)	0.16 (− 2.95, 3.37)	0.07 (− 0.32, 0.47)	−1.55 (− 7.00, 4.22)	−3.47 (− 4.27, − 2.65)	0.84 (− 4.07, 6.00)

**Fig. 1:**
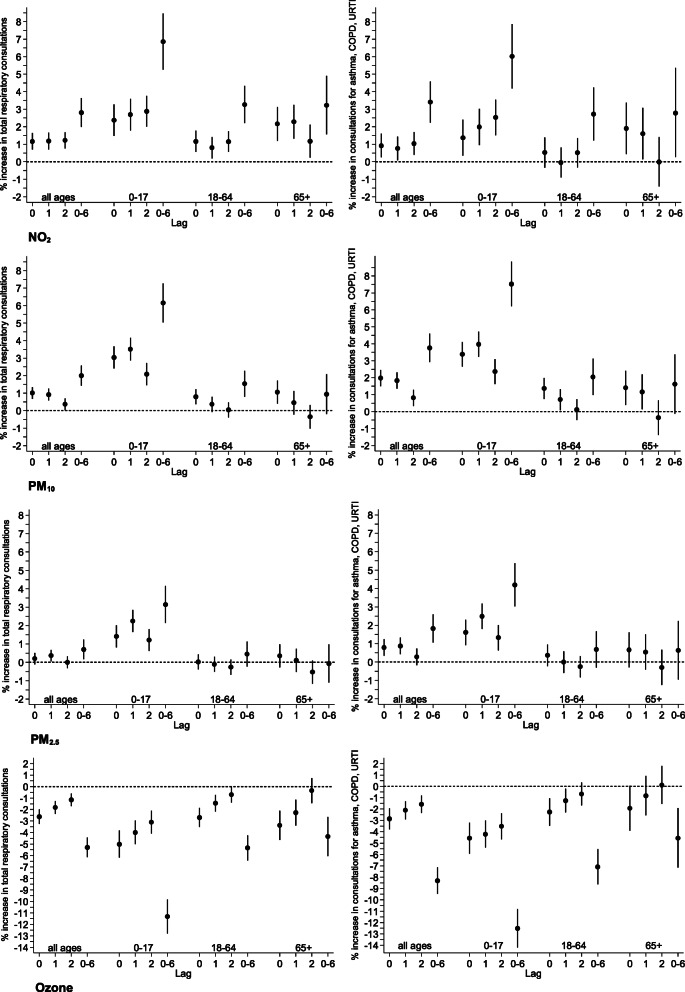
Change in the number of respiratory consultations, expressed as ‘total respiratory consultations’ and ‘asthma/COPD/URTI consultations’, (% and 95 Confidence Interval (CI)) associated with an interquartile increase in same day (lag 0), previous day (lag 1), previous 2 days (lag2) and previous week average (lag0–6) for NO_2_, PM_10_, PM_2.5_ and O_3_ concentrations, by age group.. Results from mixed effects zero-inflated negative binomial model adjusting for day of the week, temperature, relative humidity, time trend, proportion of elderly residents and index of multiple deprivation. Note: X axis values represent the lags used in days. Lag 0 estimates for each air pollutant are the same as short-term values presented in Table 3

**Fig. 2:**
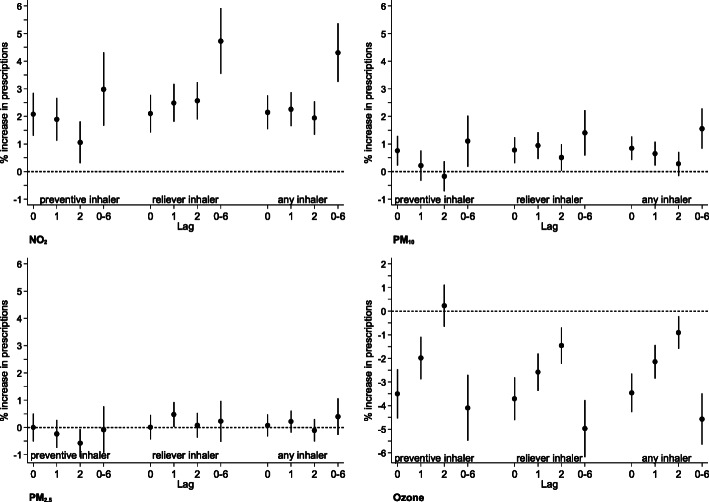
Change in the number of inhaler prescriptions (% and 95 Confidence Interval (CI)) associated with an interquartile increase in same day (lag 0), previous day (lag 1), previous 2 days (lag2) and previous week average (lag0–6) for NO_2_, PM_10_, PM_2.5_ and O_3_ concentrations. Inhaler prescription data is expressed as ‘inhaler consultations’. Note: X axis values represent the lags used in days. Lag 0 estimates for each air pollutant are the same as short-term values presented in Table 4

Figures 1 and 2 show the % changes in the number of consultations and prescriptions associated with an IQR increase in pollutant exposure for lags 0, 1, 2 and the mean of 7 days (weekly), namely lags 0–6. For NO_2_ exposure increase, the daily number of all respiratory consultations increases with 1- and 2-day lags and the association is greatest with the weekly average lag. The lag effect is particularly pronounced for 0–17 year olds where an IQR increase in NO_2_ concentrations over the previous week is associated with 6.5% increase in all respiratory consultations. For PM_10_ exposure IQR increases in 0-, 1- and 2-day lags are associated with increases in all respiratory consultations; the largest increase is associated with weekly average lag especially in 0–17 year olds (6.1%; 95%CI: 5.0 to 7.3%). Similar patterns are seen with PM_2.5_ exposure with the strongest association for weekly average lag values especially in 0–17 year olds (3.1%; 95%CI: 2.1 to 4.1%). In contrast, IQR increases in O_3_ exposure for all lags are consistently associated with decreases in the number of all respiratory consultations.

**Fig. 3:**
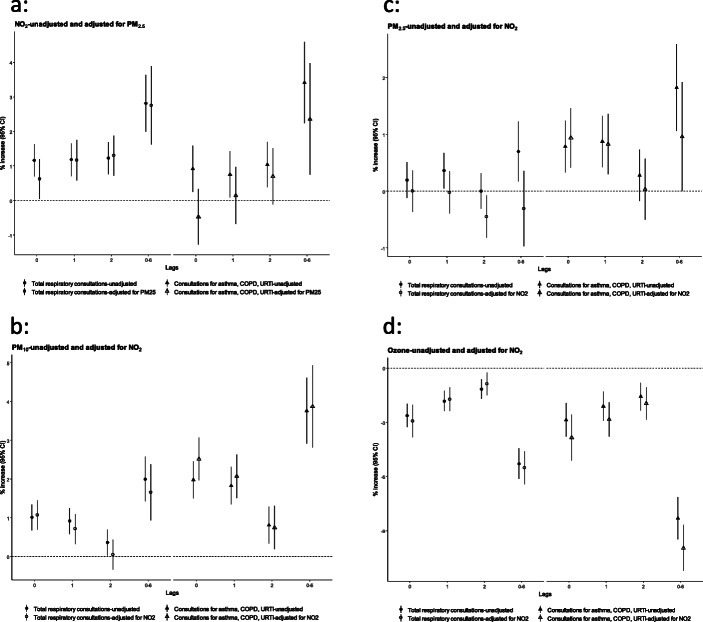
**a**-**d** Change in the number of ‘all respiratory consultations’ (% and 95 Confidence Interval (CI)) associated with an interquartile increase for NO_2_, PM_10_, PM_2.5_ and O_3_ concentrations, with same day and lag phases, adjusted for confounding by other pollutants

**Fig. 4:**
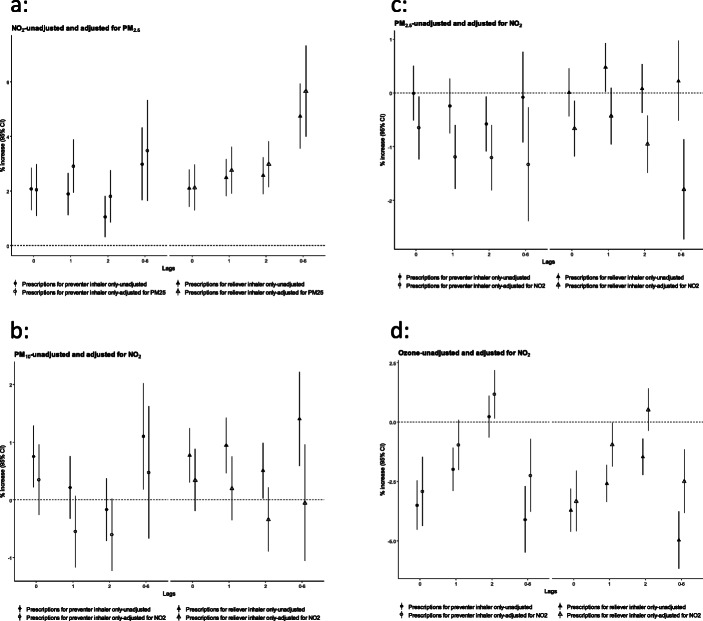
**a**-**d** Change in the number of ‘inhaler consultations’ (% and 95 Confidence Interval (CI)) associated with an interquartile increase for NO_2_, PM_10_, PM_2.5_ and O_3_ concentrations, with same day and lag phases, adjusted for confounding by other pollutants

Figures 3 and 4 shows results for short-term exposure from two pollutant models thus adjusting for possible confounding effects between pollutants. The effect estimates adjusting for another pollutant convey the same general pattern of effects even though in some instances the estimates lose statistical significance. This happens for some time lags for PM_2.5_ and PM_10_ adjusting for NO_2_. The ozone effects remain inverse and statistically significant. The ozone model was also adjusted alternatively for PM_2.5_ and the effect patterns were not changed. Effect estimates for long-term exposures do not change appreciably.

As a sensitivity analysis, we fitted Poisson time series models, as they are commonly applied in the investigation of health effects of short-term exposures [21], to assess the stability of the estimated effects of short-term exposures to pollutants. In this application, the daily pollutant concentrations were averaged over the Lambeth area. Although the pattern of the observed associations is similar, the level of statistical significance is less pronounced indicating that the use of spatially resolved data in the spatio-temporal models shown above adds some information to the analysis (data not shown).

## Discussion

In our spatio-temporal analysis of primary care data over a 5-year period (2009–13) we found that same day as well as previous 1 or 2 day or weekly average increases in NO_2_ and PM_10_ exposure are associated with significant increases in respiratory consultations, inhaler prescriptions, or both. The association was strongest for one-week average NO_2_ and PM_10_ exposure. A one quartile, one-week average increase in NO_2_ or PM_10_ was associated with approximate 3 and 4% increases respectively, in asthma/COPD/URTI consultations, and with 4 and 1.5% increases in inhaler prescriptions. When stratified by age, the strongest association was in the younger age group (0–17 years) in which one quartile, one-week average increases in NO_2_ and PM_10_ were associated with approximate 7 and 6% increases in consultations for asthma, COPD or URTI. The positive association with inhaler prescriptions was not substantially different between preventer and reliever inhalers.

Associations between PM_2.5_ exposure and respiratory consultations followed a similar pattern to those of PM_10_ exposure but were weaker. A one quartile, one-week average increase in PM_2.5_ was associated with an approximate 2% increase in asthma/COPD/URTI consultations and a 4% increase in the younger age group. However, the issuing of inhaler prescriptions was not significantly associated with PM_2.5_ exposure.

The pattern of association with ozone exposure was very different to that of the other air pollutants included in our study. Interquartile increases in ozone exposure were generally associated with reductions in respiratory consultations and inhaler prescriptions. This negative association persisted across different lag periods and age groups.

For long-term exposures, we found no statistically significant consistent associations between exposure to any pollutant and respiratory consultations, except for children where an inverse association is found. This latter result may be due to chance or to a possible residual effect of the positive association with short-term exposure. An increase in long-term exposure to NO_2_ is associated with an increase (8%) in preventer inhaler prescriptions, whilst an increase is also observed for prescriptions of preventer inhalers following long-term increases in exposure to PM_10_ and PM_2.5_, although not reaching the nominal level of statistical significance. This finding may also be due to chance, however it is noted that the use of preventer inhalers indicates a chronic condition whilst the use of reliever inhalers indicates an exacerbation and thus it is expected to see an association of long-term exposure not with indicators of an exacerbation but with indices of chronic conditions. One reason for not detecting statistically significant associations with long-term exposures may be the relatively small geographical area included in this analysis which limits the spatial contrast in pollution exposure. The clinical and prescription data analysed here are not commonly available for many boroughs. However, our findings indicate that they represent important outcomes for public health protection and it is important that such data should become available for larger areas for future work.

We applied spatio-temporal models assessing the effects of short- and long-term exposures concurrently and thus quantifying their independent effects. In the most usual types of analysis, the effects of short-term air pollution exposures are estimated by Poisson models, whilst the effects of long-term exposures are estimated by Cox proportional hazard models [17, 22]. Kloog et al. developed mixed Poisson regression models, as used in the present analysis [18]. This approach allows the counts of a health outcome by area to be modelled simultaneously as a function of both long- and short-term exposures. These models have been used previously in studies investigating the effects of air pollution exposure on mortality [17] and hospital admissions [23]. There is evidence on both the effects of short and long-term exposures of PM and NO_2_ on mortality: higher short-term exposures are associated with an acute increase in the number of deaths in a population whilst long-term exposures are associated with shorter life expectancy. There is also evidence that short-term elevations in air pollution concentrations result in higher number of hospital admissions, increased symptoms, absenteeism etc. [24–27]. Long term exposure to PM_2.5_ and O_3_ has been found to be associated with first hospital admissions for over 65’s with stroke, COPD, pneumonia, myocardial infarction, lung cancer and heart failure [28]. However, it is not entirely clear how long-term exposures affect hospital admission counts or other health events related to primary care. One plausible way is by enlarging the pool of sensitive individuals, for example those with chronic respiratory or cardiac diseases who then are more sensitive to short-term increases in air pollution. This would also result in a more pronounced short-term effect signal.

In our analysis we have used spatiotemporal modelling for primary health care data. Few previous studies analysed similar health outcomes. In the study of monthly data for an area in North-east England [4] a 10 μg/m^3^ increase in PM_10_ was associated with a 1% increase in salbutamol prescriptions. This is comparable to our finding for reliever inhaler prescriptions for a similar exposure change in 1-day PM_10_ (we used the IQR which is 9.1 μg/m^3^) and for weekly changes in adults. However, we find a much larger increase for weekly changes in prescriptions for children, whilst Sofianopoulou et al did not report analysis by age groups.

Our finding of an inverse relationship between short-term ozone elevations and the number of consultations or prescriptions was not expected and we did not find similar reports in the literature. A possible explanation may be that ozone is associated with sunny weather and high temperatures and our analysis focuses on primary care consultations; it seems plausible that good weather is associated with fewer respiratory tract infections resulting in fewer triggers to asthma or COPD exacerbation.

In our analysis, long-term exposure associations explored the spatial component of variability. In contrast to the significance of the effects of short-term exposures we do not observe significant effects of long-term exposures on GP consultations nor on overall inhaler prescriptions (with the exception of the NO_2_ association with preventer prescriptions). However, the number of daily observations used to assess the temporal variation is 1304 over a 5-year period, whilst the number of spatial units available for the analysis is smaller (*n* = 177) thus providing smaller statistical power to detect effects of long-term exposure.

All primary care data were obtained from routinely recorded consultation and prescribing activity data. Only data from coded consultations were extracted within Lambeth DataNet, thus excluding access to narrative text which may have contained additional reference to respiratory symptoms. Similarly, although almost all primary care inhaler prescribing is captured through electronic prescribing, occasional hand-written prescriptions may be issued on home visits and out-of-hours inhaler prescribing may not be transcribed into coded primary care data. Almost the entire UK population is registered with a GP (universal healthcare provision) with the exception of a few extreme socially excluded people such as the homeless. Missing data is likely to result in under-estimates of the strength of association with exposure. Nevertheless, primary health care outcome data concern a much larger proportion of the population than studies of secondary care outcomes and may be considered to be more important in terms of improving the health of communities. The main limitation of our study is the relatively restricted area coverage which was due to lack of available linked primary care health research data covering defined geographical areas, which leads to decreased power for detecting associations between spatial variability and long-term exposures. Lack of weekend data on respiratory consultations in primary care may have reduced the exposure variability and led to under-estimation of the strength of association. Additionally, the data are anonymised, thus not allowing the identification of events belonging to the same individual.

Exposure to air pollution was estimated based on LSOA of residence although working age adults are likely to be exposed to air pollutants within several LSOAs based on travel and place of work. Our study finding of stronger associations in 0–17 year olds between air pollution levels and respiratory consultations/inhaler prescriptions may be the result of increased vulnerability or confounded by lower daytime travel in this age group, especially during times of school holidays. Aggregated LSOA data is likely to underestimate the effect of individual level deprivation and may have resulted in underestimates of spatial confounding.

The present investigation became possible because of the availability of spatio-temporal models developed in the STEAM project [8–10]. These models combine a dispersion and a Land Use Regression model and, for PM_2.5_, the addition of satellite data and machine learning methods. They predict on a daily basis and provide estimates per LSOA (based on an average of predictions for all post-code centroids included in an LSOA). It is evident that even the most dense fixed site monitoring network cannot provide an adequate spatial resolution for such a spatiotemporal health analysis. Hybrid models open the way for more powerful and sophisticated analyses leading to a better understanding of health effects.

These detailed spatio-temporal models had a stronger predictive ability at the temporal rather than the spatial scale. For example, the PM_2.5_ model has a spatial R^2^ equal to 0.40 and a temporal R^2^ of 0.88 [10]. This limits the interpretation of respiratory health associations with the spatial component of PM_2.5_ variability in our analysis. European air pollutant values are atypical in some respects. Higher usage of light duty diesel vehicles and differences in heavy industry in European countries results in relatively low PM and high NO_2_ levels compared to non-European contexts such as Asian and North America [29, 30]. Future work is needed to improve the prediction of the spatial variability component and to develop such models for other geographical areas, as the consequences of air pollution have to be considered in a global context.

A further limitation of our study is that we use aggregated data, albeit data aggregated at a very fine spatial level, and thus we are not able to include information on individual confounders in the models. We have included only LSOA level confounders, specifically age distribution and deprivation index. It is possible that this level of adjustment does not fully control for the relevant confounders.

## Conclusion

In conclusion, our study is one of the few to investigate the associations of short- and long-term exposure to ambient PM, NO_2_ and ozone air pollutants with commonly occurring primary care respiratory consultations and the prescribing of respiratory inhalers.

Short term increases in PM and NO_2_ are associated with increased asthma, COPD and URTI consultations for all ages. These associations are most pronounced in children and with sustained, one-week elevations in air pollutants. Similar for NO_2_ but less strong for PM, associations are also seen with both preventer and reliever inhaler prescriptions.

Higher levels of ozone exposure were associated with lower rates of respiratory consultations and inhaler prescriptions, possibly the result of known associations between high ozone levels and hot and sunny weather.

There is a need to for more studies investigating air pollutants exposure over sustained periods with primary care data covering wider geographical areas and larger populations.

## Data Availability

Under the terms of the data sharing agreement (see above), original patient level data is not available to external researchers.
